# *In Vitro* Antimicrobial Activity of Ethanolic Extract of Polish Propolis against Biofilm Forming *Staphylococcus epidermidis* Strains

**DOI:** 10.1155/2013/590703

**Published:** 2013-04-10

**Authors:** Robert D. Wojtyczka, Małgorzata Kępa, Danuta Idzik, Robert Kubina, Agata Kabała-Dzik, Arkadiusz Dziedzic, Tomasz J. Wąsik

**Affiliations:** ^1^Department and Institute of Microbiology and Virology, School of Pharmacy and Division of Laboratory Medicine in Sosnowiec, Medical University of Silesia, Katowice, Jagiellońska 4, 41-200 Sosnowiec, Poland; ^2^Department and Institute of Pathology, School of Pharmacy and Division of Laboratory Medicine in Sosnowiec, Medical University of Silesia, Katowice, Poland; ^3^Department of Conservative Dentistry with Endodontics, Medical University of Silesia, Katowice, Poland

## Abstract

The aim of the presented study was to examine the antimicrobial activity of ethanol extract of Polish propolis (EEPP) against biofilm-forming CoNS strains *in vitro*. Our results revealed that EEPP displayed varying degrees of activity against CoNS with MIC values ranging from 1.56 to 0.78 mg/mL. The average MIC was 1.13 ± 0.39 mg/mL while calculated MIC_50_ and MIC_90_ values were 0.78 mg/mL and 1.56 mg/mL, respectively. The biofilm formation ability by all tested *S. epidermidis* strains was inhibited at EEPP concentrations ranging from 0.39 to 1.56 mg/mL. The degree of reduction of AlamarBlue was directly associated with the proliferation of *S. epidermidis *strains. The increased proliferation of *S. epidermidis *strains was observed after 12 and 24 hours of incubation in the presence of EEPP concentrations ranging from 0.025 to 0.39 mg/mL. These results suggest that antimicrobial activities of EEPP against *S. epidermidis* expressed as the reduction of bacterial growth, reduction of biofilm formation ability, and the intensity of proliferation were significantly affected by incubation time and EEPP concentration used as well as the interactions between these factors.

## 1. Introduction

Staphylococci species are differentiated by the ability to express coagulase, an enzyme that enables the conversion of fibrinogen to fibrin, for example, *S. aureus*, *S. intermedius*, *S. delphini*, and some strains of *S. hyicus* and *S. schleiferi*. Staphylococci that do not produce coagulase are referred to as coagulase-negative staphylococci (CoNS) [1, 2]. Clinically, the most significant species in this group are *S. epidermidis* and *S. saprophyticus*, which have been known to be responsible for a variety of hospital-acquired infections and to be associated with development of urinary tract infections [3].

*S. epidermidis*, a microorganism routinely found on the skin and in the hospital environment [4, 5], often described as a culture contaminant [6, 7], has become a primary pathogen in infections associated with the prosthetic devices. *S. epidermidis* is considered to be an important pathogen in immunocompromised individuals with surgical wound infections or bacteremia and persons who developed nosocomial bacteremia [8, 9]. It may be also responsible for many infections associated with hemodialysis, a long-term indwelling of central catheter or pacemaker, or other invasive procedures with the use of medical implants [10]. CoNS have recently emerged as an important causative factor in the native valve endocarditis (NVE) development. Most cases of NVE caused by CoNS are attributable to *S. epidermidis* in both community and health care settings and are related to the poor treatment outcomes [11]. Since the majority of *S. epidermidis *infections, except NVE are hospital-acquired, the poor prognosis of NVE may require the need for alternative therapies with efficient activity against methicillin-resistant CoNS [12]. 

CoNS due to their variability and relatively low virulence are often misidentified. Resistant antibiotic susceptibility pattern of *S. epidermidis* may cause selection of an effective antibiotic regimen extremely difficult [13]. Strains isolates from nosocomial infections are frequently resistant to methicillin and other synthetic antibacterial agents. The identification of CoNS is routinely performed with the use of the diagnostic kits based on biochemical or immunological reactions. However, they are unreliable for the identification of CoNS species including *S. epidermidis* [14]. Up to date a ribosomal RNA (rRNA) method-based analysis is the best and the most reliable method for the phylogenetic determination within CoNSs and species identification [15, 16]. The application of restriction fragment length polymorphisms (RFLP) of rRNA genes for differentiation of clinical isolates of *S. epidermidis *seems to be a highly specific and reliable modern method for molecular identification of these bacteria [17].

Propolis, a resinous substance produced by honeybees, has been used by humans as a remedy in traditional medicine for its health properties since ancient times, and it is still used for treatment of wounds, burns, sore throat, and so forth [18]. Propolis contains various chemical components, which exhibit a broad spectrum of biological activities [19]. The composition of propolis is complex and largely depends on the geographical origin and specific flora at the site of its collection [20–22]. Numerous researches have been carried out to identify and characterize the antibacterial and antifungal compounds of propolis. Phenolic substances, flavonoids, and cinnamic acids derivatives compose the major bioactive components of propolis [23–25]. The antimicrobial proprieties of propolis are related to the synergistic effect of its components [26]. It has been demonstrated that EEP exhibits a wide range of biological activities, including bacteriostatic activity against many strains with a significant effect on Gram-positive and a limited action on Gram-negative bacteria [27–29]. However, there are only few study reports published where effects of propolis against biofilms-forming coagulase-negative staphylococci or multidrug resistant pathogens were investigated. It was found that ethanol extracts of propolis can inhibit growth of the multidrug resistant bacteria, such as methicillin-resistant *S. aureus *(MRSA), *Enterococcus* spp., and *Pseudomonas aeruginosa* [30]. A study on the effect of EEP from Turkey against 39 microorganism, including resistant or multidrug resistant, demonstrated significant antimicrobial activities against Gram-positive bacteria and yeasts [31]. Furthermore, it has been revealed that propolis could synergize the antimicrobial effect with selected antimicrobial drugs against *S. aureus *especially those agents that interfere with the bacterial protein synthesis [26]. It is believed that the ability to form biofilms on the surfaces of medical implants is one of the most important virulence factor of *S. epidermidis* [32]. The formation of the polysaccharide intercellular adhesin (PIA) allows planktonic bacteria to bind to the already existing biofilm, thus creating a multilayers [33], which in turn, with decreased metabolism and in combination with impaired diffusion of antibiotics, is responsible for diminishing drug efficacy in fighting this type of infection [34]. Since many reports showed that antibiotics were often ineffective in biofilms eradication, further studies regarding biological anti-CoNS agents may support the need for alternative antibacterial protocols to be applied for the treatment of nosocomial infections caused by *S. epidermidis*. 

The purpose of this work was to assess the effective inhibitory and bactericidal concentration of EEP of the Southeastern Poland origin against biofilm-forming CoNS, identified by PCR-RFLP molecular technique under *in vitro* conditions.

## 2. Material and Methods

### 2.1. Bacterial Strains

The antibacterial activity of EEPP was assessed against 10 CoNS strains isolated from clinical blood samples and *S. epidermidis* ATCC 35983 as the biofilm positive control. Isolates were identified by conventional methods, including Gram staining, colony morphology, hemolysis, tests for catalase, coagulase activity, and anaerobic fermentation of mannitol. Catalase-positive and coagulase-negative staphylococcal isolates were identified by the API STAPH system (bioMerieux) according to the manufacturer's instructions. The PCR-RFLP molecular methods were used for CoNS species identification. 

Bacterial strains isolated from clinical samples were stored for further analyses in TSB (Trypticase Soy Broth) medium with 20% of glycerol at −86°C. 

### 2.2. PCR-RFLP Analysis of DnaJ Gene

To confirm the correct identification of staphylococci strains by standard microbiological methods, PCR-RFLP method described previously by Shah et al. was used. Briefly, the *dnaJ *primers SA-(F) (5′-GCC AAA AGA GAC TAT TAT GA-3′) and SA-(R) (5′-ATT GYT TAC CYG TTT GTG TAC C-3′) were used to amplify the *dnaJ *gene fragment [35]. The PCR reactions were performed using 10xPCR RED master mix kit (BLIRT S.A. Poland). PCR was performed using an MJ mini personal thermal cycler (Bio-Rad). The PCR products were separated and visualized in 1.5% agarose gel (PROMEGA) with ethidium bromide (EtBr) and checked for size against molecular weight markers using 1 Kb HyperLadderIV (BLIRT S.A., Poland). 

In order to identify isolated staphylococci strains we used the species-specific restriction profiles obtained by using *XapI *or *Bsp143I *restriction enzymes. Digestions were performed with 5 *μ*L of the PCR products in a total volume of 15 *μ*L with 1 *μ*L of reaction buffer and either 10 U of the *XapI* endonuclease or 10 U of the *Bsp143I* endonuclease (Fermentas, Lithuania) for 3 hours at 37°C [36]. The obtained fragments were separated by electrophoresis in 2% agarose gels (PROMEGA) and visualized under UV light after EtBr staining.

### 2.3. Detection of icaA, icaD, icaB, and icaC

The bacterial DNA was isolated using Genomic DNA Mini Kit (BLIRT S.A., Poland). Briefly, strains stored at −86°C were thawed at room temperature, subcultured on blood agar plates, and checked for their purity prior to DNA isolation. Cultured bacteria were suspended in 100 *μ*L of TRIS buffer with 10 *μ*L of lysostaphin (1 mg/mL; BLIRT SA, Poland) and incubated at 37°C for 30 minutes. The suspensions were treated with proteinase K and LT buffer and incubated at 37°C overnight with final incubation at 75°C for 5 minutes. DNA was purified according to the protocol, using ethanol and washing buffer supplied in the kit, suspended in 200 *μ*L of TRIS buffer, and stored at −20°C for further analyses.

A standard PCR technique was used to detect the presence of *icaA, icaD, icaB, *and* icaC *genes previously described by Ziebuhr et al. [37] and de Silva et al. [38]. The primer sequences for *icaA* were 5′-GAC CTC GAA GTC AAT AGA GGT 3′ (forward) and 5′ CCC AGT ATA ACG TTG GAT ACC 3′ (reverse); *icaD:* 5′AGG CAA TAT CCA ACG GTA A3′ (forward) and 5′-GTC ACG ACC TTT CTT ATA TT-3′ (reverse); *icaB:* 5′ ATA AAC TTG AAT TAG TGT ATT 3′ (forward) and 5′ ATA TAT AAA ACT CTC TTA ACA 3′ (reverse); and *icaC:* 5′ AGG CAA TAT CCA ACG GTA A 3′ (forward) and 5′ GTC ACG ACC TTT CTT ATA TT 3′ (reverse).

PCR was performed using an MJ mini personal thermal cycler (Bio-Rad, CA, USA). The PCR products were visualized in agarose gels with EtBr and checked for size against molecular weight markers using 1 Kb HyperLadderIV (BLIRT S.A., Poland).

### 2.4. Microtiter Plate Assay (TCP)

To analyze biofilm formation by isolated staphylococci, the method described by Christensen et al. [39] with modifications was used. Bacteria were suspended in Muller-Hinton Broth (MHB-BTL, Poland) giving the cell density equal to 0.5 of the McFarland standard. 100 *μ*L of each bacterial suspension was inoculated into 96-well microtiter plates. The plates were incubated at 37°C for 24 hours in a normal atmosphere. Next, medium was removed, and the wells were washed three times with phosphate saline buffer (PBS, pH = 7.2) to remove free floating “planktonic” bacteria. Next, 150 *μ*L of 1% crystal violet (Sigma) was added into each well and incubated for 30 minutes at room temperature. The dye was removed, by five times washing with sterile deionized water. The samples were incubated with 200 *μ*L of 95% isopropanol in 1 M HCl for 5 minutes. Finally, 100 *μ*L of colored isopropanol from each sample was transferred to another microtiter plate. The optical density of suspension was measured at 490 nm wave length (A_490_) with a Multitec SX microplate reader. The assay was conducted in triplicates and mean A_490_ ± SD values were calculated. The values of optical density for samples were compared with those obtained for negative control (wells without bacterial inoculum). According to Christensen et al. [39] the samples with the A_490_ >0.11 were considered as positive. In the presented study bacterial strains were considered as nonadherent when their optical density was equal to or lower than 0.11, weakly adherent when optical density was higher than 0.11 or equal to or lower than 0.17, and strongly adherent when optical density was higher than 0.17.

### 2.5. Antibacterial Susceptibility Testing

MICs of EEPP were determined by the broth microdilution liquid growth inhibition method. Growth inhibition assays were performed with sterile Nunc 96-well plates in a final volume of 200 *μ*L [40, 41]. The cell concentrations were estimated from the optical densities at 600 nm with the formula CFU/mL = A_600_ (3.8 × 10^8^), where CFU is the number of colony-forming units. One hundred microliters of midlogarithmic-phase bacterial cultures (5 × 10^5^ CFU/mL) in Mueller-Hinton broth was added to 100 *μ*L of serially diluted EEPP (12.5 to 0.02 mg/mL). Samples comprising bacterial inoculum without EEPP were reserved as the bacterial growth and medium sterility control. The control of activity of ethanol alone without propolis towards *S. epidermidis* ATCC 25883 strain was performed. The microplates were incubated at 37°C for 20 hours, and the bacterial cell growth was assessed by measuring the optical density of cultures at 600 nm with a Multiskan EX microplate reader (Thermo Electron Corp., Finland) [42, 43]. 

The MICs were recorded as the lowest concentration that completely inhibited bacterial growth [40–42]. The MIC_50_ represents the MIC value at which ≥50% of the isolates in a test population are inhibited; it is equivalent to the median MIC value. The MIC_90_ represents the MIC value at which ≥90% of the strains within a test population are inhibited, the 90th percentile [44].

### 2.6. AlamarBlue Susceptibility Assay

Antimicrobial susceptibility testing of planktonic forms of the biofilm-forming *S. epidermidis* strains was performed by the reference broth microdilution assay, using round-bottom, polystyrene, nontissue, and culture-treated microtitre plates and Muller-Hinton II Broth according to the manufacturer procedure (USA Patent no. 5,501,959). The bacterial cultures were prepared as described above and incubated for 20 hours at 37°C; next, 5 *μ*L of AlamarBlue was added into each well (105 *μ*L total volume), the plates were shaken gently and incubated for 2 hours at 37°C. The absorbances at 570 nm and 600 nm wave lengths were measured using a Multiskan EX microplate reader (Thermo Electron Corp., Finland).

Four different controls were used in this experiment, that is, medium only, medium with AlamarBlue reagent (AB), medium with AB reagent and different propolis concentrations, and medium with cells and AB reagent. The reduction of bacterial proliferation (%AB) was calculated according to the manufacturer's formula. The values of %AB reduction were corrected for background values of negative controls containing medium without cells.

The assay was performed in three replicates for two different experiments. AlamarBlue MIC (MIC_AB_) was defined as the lowest EEPP concentration resulting in ≤50% reduction of AB [45].

### 2.7. Statistical Analyses

The data obtained for bacterial growth were analysed by a three-way analysis of variance (ANOVA) to determine the percentage of the variation attributable to the factors bacterial strains, time, and concentrations. All statistical analyses were made using the Statistica 10.0 PL software package.

## 3. Results

**Figure 1:**
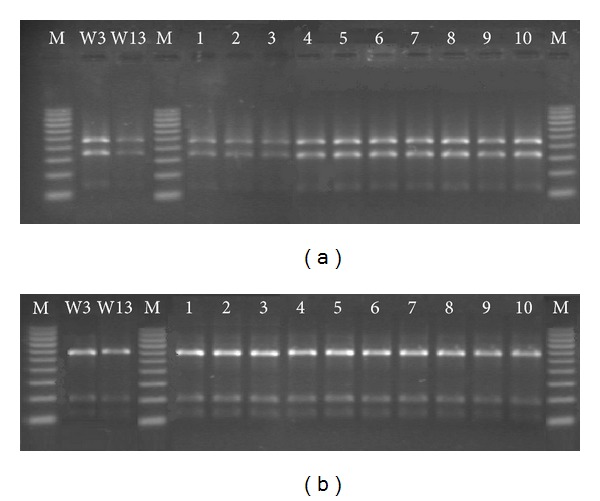
Restriction fragment length polymorphism analysis of *dnaJ* gene fragment digested with *XapI* (a) and *Bsp143I *(b). W3—*S. epidermidis* ATCC 12228; W13—*S. epidermidis* ATCC 35983; 1–10 *S. epidermidis* strains selected for further experiment; M—100–1000 bp marker.

The molecular species identification of CoNS by PCR-RFLP technique with *XapI *and* Bsp143I* restriction enzymes confirmed that all isolated strains were found to be as *S. epidermidis* (Figures 1(a) and 1(b)). 

Molecular analysis revealed that 10* S. epidermidis *strains carried *icaA* gene while *icaD* was present in 8, *icaB* in 11, and *icaC* in 10 strains. Six strains carried all genes from *icaADBC* operon (Table 1).

The biofilm formation ability by bacteria was assessed by TCP method. The applied method showed that optical densities of all analyzed *S. epidermidis* cultures at 490 nm wave length (A_490_) were greater than 0.11 and varied from 0.12 to 3.85. Two strains showed a relatively low biofilm formation ability with A_490_ ranged from 0.11 to 017 while the remaining strains and reference strain *S. epidermidis* ATCC 35983 showed a good biofilm formation ability with A_490_ values higher than 0.17.

The broth microdilution method was used to determine the MIC of the EEPP against 11 CoNS. EEPP displayed varying degrees of activity against CoNS with MIC values in the range of 1.56–0.78 mg/mL (Table 2). The average MIC was 1.13 ± 0.39 mg/mL while calculated MIC_50_ and MIC_90_ values were 0.78 mg/mL and 1.56 mg/mL, respectively. 

**Figure 2:**
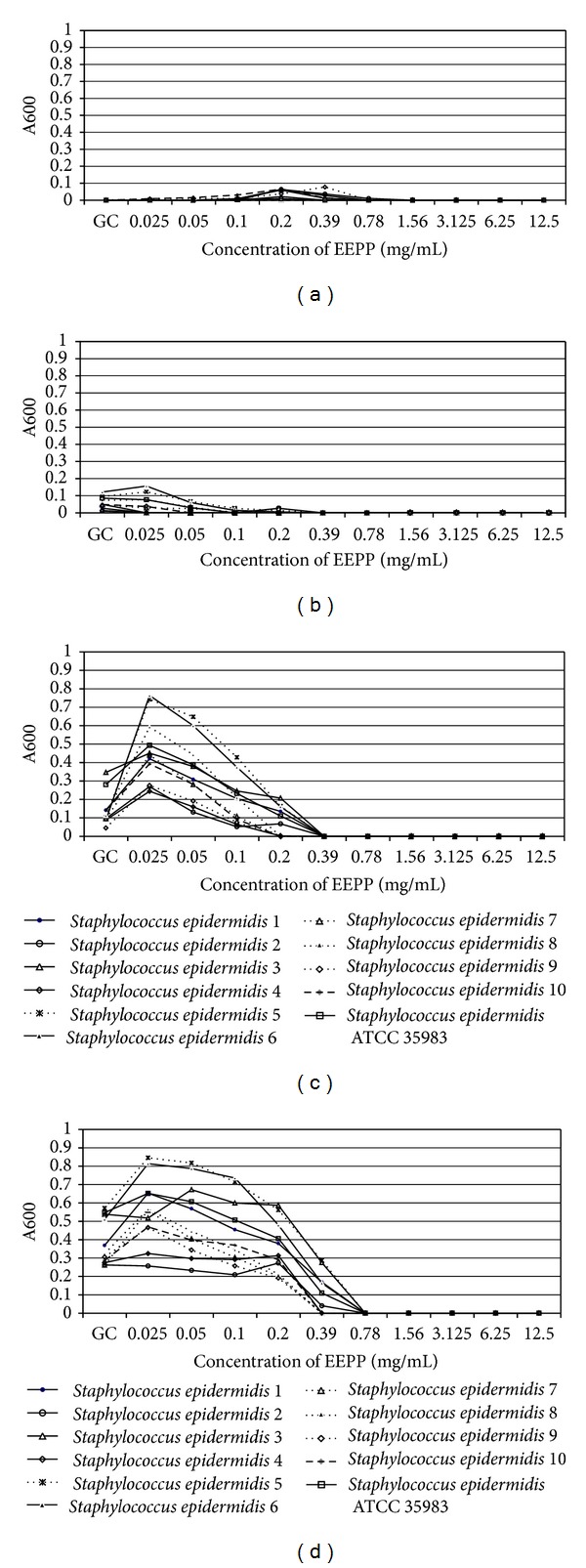
Growth kinetics of *S. epidermidis *strains in the presence of different EEPP concentrations. (a) After 2 hours of incubation; (b) after 6 hours of incubation; (c) after 12 hours of incubation; and (d) after 24 hours of incubation.

The analysis of growth kinetics after the first two hours of incubation showed a similar growth pattern for *S. epidermidis *strains cultured in medium with different EEPP concentrations and in medium without EEPP (Figure 2(a)). After 6 hours of incubation, the growth of all strains was observed in medium supplemented with EEPP at concentrations ranging from 0.025 to 0.39 mg/mL, and in addition, the growth of *S. epidermidis* strains in medium with two lower EEPP concentrations was similar to the growth control (Figure 2(b)). After prolonged incubation time (12 and 24 hours) tested *S. epidermidis *strains revealed differences in susceptibility to EEPP used at concentration ranging from 0.025 to 0.78 mg/mL (Figures 2(c) and 2(d)).

The ANOVA indicated that the growth kinetics of all biofilm-forming *S. epidermidis* strains was significantly affected by EEPP concentration (*P* < 0.001) and incubation time (*P* < 0.001). The interaction between these factors was also significant (*P* < 0.001). The EEPP conentration effect (83.88%) and interaction between concentration and incubation time (10.94%) explained most of variance (Table 3). 

**Figure 3:**
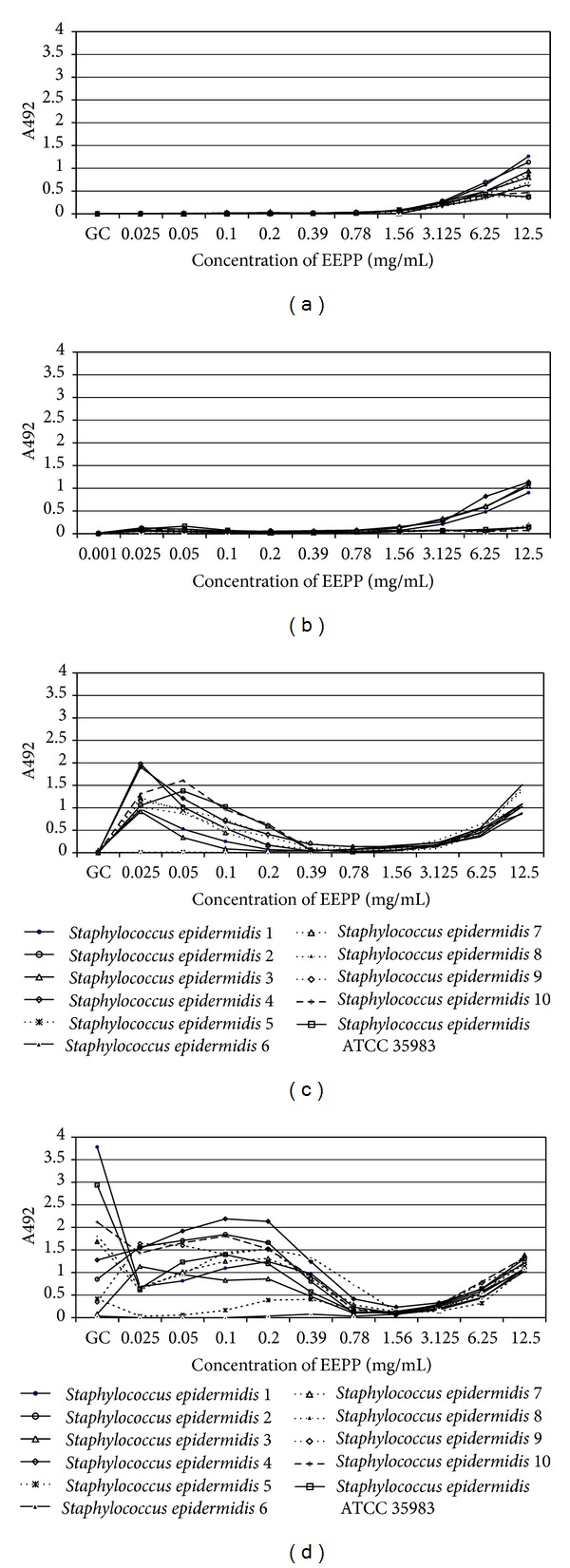
The biofilm formation ability of *Staphylococcus epidermidis *strains in the presence of different EEPP concentrations. (a) After 2 hours of incubation; (b) after 6 hours of incubation; (c) after 12 hours of incubation; and (d) after 24 hours of incubation.

The biofilm formation ability by all tested *S. epidermidis* strains was inhibited at EEPP concentrations ranging from 0.39 to 1.56 mg/mL (Figure 3). This effect was observed after 12 hours of incubation at EEPP concentration greater than 0.2 mg/mL. Interestingly, EEPP at concentrations lower than 0.025 mg/mL seemed to be the factor increasing the biofilm formation ability as compared to the control after 12 hours of incubation (Figure 3(c)). The ANOVA indicated that the biofilm formation ability by all *S. epidermidis* strains in the presence of EEPP was significantly affected by incubation time (*P* < 0.001), the interaction between incubation time, and EEPP cocentration (*P* = 0.032). However, the interaction between incubation time, EEPP concentration, and bacterial strain explained most of variance (26.78%) (Table 4). 

**Figure 4:**
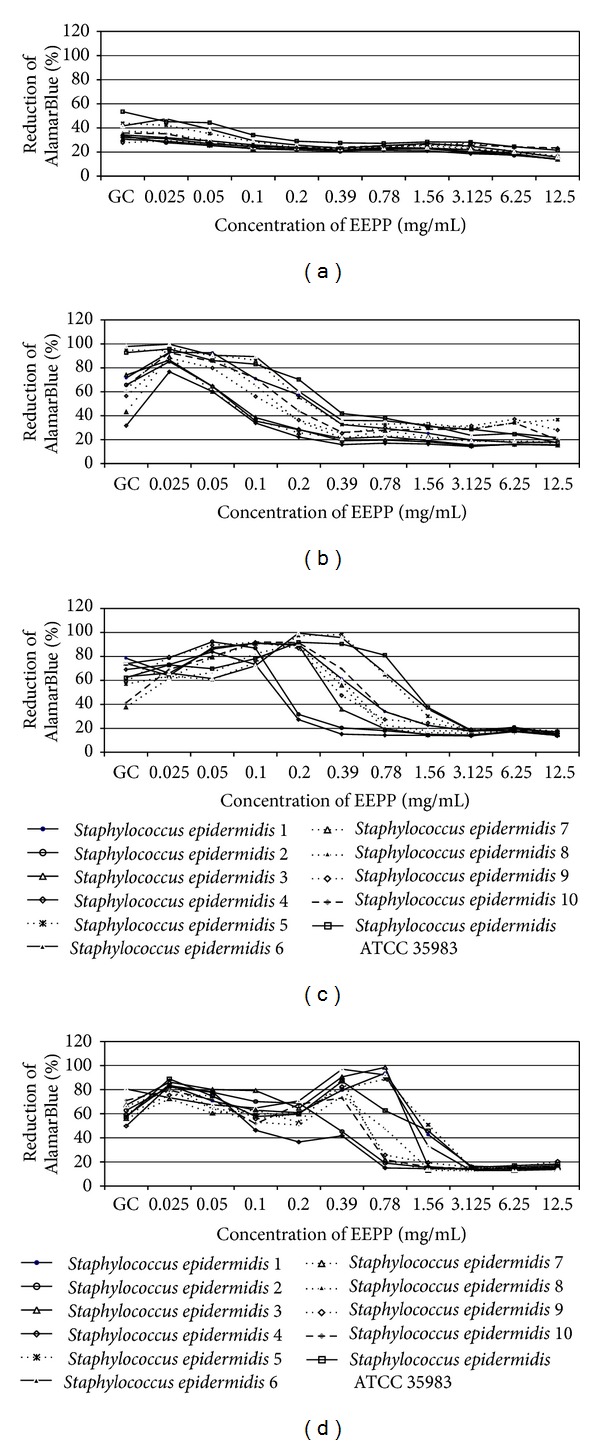
The AlamarBlue reduction ability of *S. epidermidis *strains in the presence of different EEPP concentrations. (a) After 2 hours of incubation; (b) after 6 hours of incubation; (c) after 12 hours of incubation; and (d) after 24 hours of incubation.

The degree of AlamarBlue reduction is directly associated with the proliferation of *S. epidermidis *strains. Analysis of absorbance changes revealed that the first effect of EEPP on bacterial proliferation was observed after 2 hours of incubation (Figure 4(a)). After 6 hours of incubation the proliferation of bacterial strains was stimulated at lower EEPP concentrations (0.025–0.05 mg/mL). The increased proliferation of *Staphylococcus epidermidis *strains was also observed after 12 and 24 hours of incubation in the presence of EEPP at concentrations ranging from 0.025 to 0.39 mg/mL (Figures 4(c) and 4(d)) while at higher EEPP concentrations proliferation was significantly diminished. The MIC_AB_ ranged from 0.2 to 1.56 mg/mL.

The ANOVA indicated that the proliferation of all strains in the presence of EEPP was significantly affected by incubation time (*P* < 0.001), EEPP concentration (*P* < 0.001), and strain (*P* < 0.001), and the interactions between all these factors were also significant (*P* < 0.001). The EEPP concentration (29.73%), time (24.94%), and interactions between these factors (28.99%) explained most of variance (Table 5). 

The biological activity of EEPP seen in the present study was not influenced by the ethanol presence in the EEPP solutions for no effect of ethanol solution, free of Polish propolis, on the *Staphylococcus epidermidis* ATCC 35893 strain was observed, data not shown.

## 4. Discussion

Several studies have demonstrated that propolis might exert diversified effects on many bacterial strains. Mantovani et al. [46] demonstrated strong anti-CoNS activity of propolis. Najmadeen and Kakamand studies [47] on ethanol extracts of propolis activities on *S. epidermidis* and *S. aureus *showed that *S. epidermidis* strains were less susceptible to different propolis extracts than coagulase positive staphylococci. It has been shown that EEPP possesses antibacterial activity against different Gram-positive bacterial strains, including *S. epidermidis* [48]. The EEP biological activity against *S. epidermidis* may vary significantly with respect to different propolis sources and extract types [49]. According to Najmadeen and Kakamand [47], propolis might be even more efficient than some antibiotics for inhibition of bacterial growth and proliferation. In the disc diffusion method with the standardized propolis extract the most susceptible bacteria toward EEP, with mean inhibitory diameters (22–26 mm), was *S. epidermidis* followed by *S. aureus *and *Candida albicans *(15–22 mm). In turn, results of MIC and MBC showed that the most sensitive bacteria was *S. aureus *(0.175–0.7 mg/mL) followed by *S. epidermidis* and *C. albicans *(0.7–1.4 mg/mL) [47]. In the present study, the biofilm-forming *S. epidermidis* strains showed MIC_EEPP_ values ranging from 0.78 to 1.56 mg/mL, which was in agreement with MICs obtained by Najmadeen and Kakamand [47]. Interestingly, in this study all *S. epidermidis* strains MIC_EEPP_ ranged from 0.2 to 0.39 mg/mL and from 0.39 to 0.78 mg/mL after 12 and 24 hours of incubation, respectively, which suggested that EEPP activity might diminish over time. The determination of the chemical characteristics of EEP showed that the phenolic compounds were mainly responsible for the anti-CoNS activity of EEP collected from the Southeast of Brazil [46]. The findings of another study, evaluating the antibacterial properties of extracts of propolis from Mexico, revealed that the highest sensitivity towards propolis was shown by *S. aureus*, *S. epidermidis,* and the two *Vibrio* cholerae strains with MICs values <0.125 mg/mL [49]. The results presented by Pinto et al. [50] showed that propolis and its ethanolic extract inhibited growth of the Gram-positive bacteria, *S. aureus*, CoNS, and *Streptococcus agalactiae*. 

Berretta et al. [51] concluded that microorganisms, such as *Pseudomonas aeruginosa*, *Klebsiella pneumoniae*, *Escherichia coli*, *S. aureus, *and *S. epidermidis *are the most frequently isolated from injuries and burn wounds. The authors tested antimicrobial activity of the different extracts and propolis formulations against the above-mentioned microorganisms and concluded that antimicrobial and wound-healing activity showed the best results when applying pharmaceutics containing 3.6% addition of propolis. 

The mechanism of propolis antibacterial activity seems to be linked to some of its constituents. The potent bacteriostatic and bactericidal effects of propolis are the result of the combined actions of several such components. Oksuz et al. [52] and Havsteen [53] suggested that the specific propolis ingredients inhibit protein synthesis and bacterial growth by preventing cell division, resulting in the formation of pseudomulticellular bacterial forms. Galangin and caffeic acids from EEP are enzymatic inhibition agents responsible for an inhibition of bacterial growth and proliferation. In addition, some active substances composing propolis may disorganize the cytoplasmic membrane and cell wall, with the effect of a partial bacteriolysis. Flavonoids affect bacterial membrane potential and cause permeability alteration within the inner microorganisms membrane [54]. Takaisi-Kikuni and Schilcher [55] revealed that the inhibition of bacterial RNA-polymerase by the components of propolis was probably associated with the loss of their ability to bind to DNA. It is believed that antimicrobial and anti-inflammatory properties of propolis are mainly attributed to its flavonoid and phenolic compounds composition [51, 56]. Some of these biomolecules, such as galagin and caffeic acids, are considered to be bacterial enzymes inhibitors [53].

It has been shown that biofilm formation by some bacteria is one of the important microbial defense strategies against xenobiotics. Stewart and Costerton [57] suggested three different mechanisms associated with antibiotics resistance of some bacterial strains producing biofilm: (i) biofilm matrix causes an incomplete antibiotic penetration; (ii) the chemical composition of biofilm microenvironment connected to bacterial metabolism protects cells; (iii) in biofilm subpopulation of microorganisms can grow in a unique, highly protected phenotypic forms, in which the cells gain features of spores. In this study, the inhibitory effect on biofilm formation by the majority of tested *S. epidermidis* strains in the presence of EEPP was observed after 12 and 24 hours of incubation, and it was correlated with MIC values. Interestingly, after 12 hours of incubation at lower EEPP concentrations, the transient, reverse effect was observed suggesting stimulatory effect of propolis on biofilm formation. 

The observed growth kinetics of *S. epidermidis *in subsequent hours of the experiment showed growth stimulation at the low concentrations of EEPP, mainly after 12 and 24 hours. This effect could be caused by the presence of the nutrients in EEPP acting as a growth stimulators. This phenomenon was associated with the acceleration of biofilm formation. However, the bacteria cells survival rate of the planktonic forms, assessed by the AlamarBlue assay, was reduced. The results showed that EEPP affected essentially the planktonic forms of biofilmforming* S. epidermidis*. This observation may suggest that EEPP affects the planctonic forms of bacteria with the ability to form biofilm rather than the architecture of the biofilm itself. 

*S. epidermidis* strains are often resistant to antibiotics, including penicillin, amoxicillin, and methicillin. Most of *S. epidermidis* isolates are susceptible *in vitro* to vancomycin and rifampicin. However, Penicillin G, semisynthetic penicillinase-resistant penicillins, and cephalosporins are effective for the treatment of methicillin-sensitive *S. epidermidis* infections [58]. Studies on the possible synergism between propolis (collected in Brazil and Bulgaria) and antibiotics (chloramphenicol, tetracycline, and neomycin) showed that Bulgarian propolis had antibacterial action, as well as a synergistic effect with antibiotics acting on the ribosome [59]. These observations were further confirmed by other authors [60, 61].

The analysis of proliferative properties of *S. epidermidis* strains in the presence of EEPP expressed as percent of reduction of AlamarBlue revealed that this reduction for MIC values 0.78–1.56 mg/mL was at a level of 18–80%. In addition, this process was strain specific. Similarly, reduction of bacterial proliferation was observed for higher EEPP concentrations while at lower concentrations ranging from 0.025 to 0.1 mg/mL propolis seemed to accelerate proliferation of some strains.

## 5. Conclusion

Propolis belongs to the natural antimicrobial agents, which in many studies has shown to be fairly effective and promising treatment of serious Gram-positive infections, including the hospital-acquired infections caused by CoNS and multiresistant strains. The application of the EEPP on the skin and/or oral mucosa as a preoperative prophylactic protocol may prevent the potential infection by reducing the* S. epidermidis* colonies's growth. The results presented in this study suggest that antimicrobial activity of EEPP against *S. epidermidis* expressed as the reduction of bacterial growth and biofilm formation ability as well as the intensity of proliferation is time and concentration dependent. The observed transient increase of biofilm formation ability in the presence of propolis at low concentrations requires further study. Microbiota characterized by biofilm formation ability represents the increased resistance to antibacterial drugs; therefore, there is a great need to continue research regarding the development of the new substances which may support the elimination of these microorganisms.

## Figures and Tables

**Table 1:** The biofilm formation ability and genetic composition of *S. epidermidis* strains.

Strain	*icaA *	*icaD *	*icaB *	*icaC *	TCP A_490_
1	+	+	+	+	3.85
2	+	+	+	−	0.91
3	+	−	+	+	0.15
4	+	+	+	+	1.34
5	+	+	+	+	0.52
6	+	+	+	+	0.12
7	+	−	+	+	1.78
8	−	+	+	+	1.92
9	+	+	+	+	0.45
10	+	−	+	+	2.21
K*	+	+	+	+	3.08

K*: biofilm-forming* S. epidermidis* ATCC 35983.

**Table 2:** Susceptibility of *S. epidermidis* strains to EEPP (MICs in mg/mL).

Strain	MIC EEPP (mg/mL)
1	1.56
2	0.78
3	1.56
4	0.78
5	1.56
6	0.78
7	1.56
8	0.78
9	0.78
10	0.78
K*	1.56

K*: biofilm-forming* S. epidermidis* ATCC 35983.

**Table 3:** Multivariate analysis of variance by three-way ANOVA of *S. epidermidis* strains susceptibility to EEPP.

Source of variation	df	Sum of squares	Mean squares	% of variance	*F*	*P*
Strain (*S*)	10	1.97	0.2	0.59	127.64	<0.001
Time (*T*)	3	3.45	1.51	1.04	744.49	<0.001
Concentration (*C*)	10	277.57	27.76	83.88	17946.44	<0.001
*S* × *T*	30	2.55	0.09	0.77	55.12	<0.001
*S* × *C*	100	5.32	0.05	1.61	34.42	<0.001
*T* × *C*	30	36.19	1.21	10.94	780.04	<0.001
*S* × *T* × *C*	300	3.11	0.01	0.94	6.70	<0.001

**Table 4:** Multivariate analysis of variance by three-way ANOVA of *S. epidermidis* strains biofilm formation ability in the presence of EEPP.

Source of variation	df	Sum of squares	Mean squares	% of variance	*F*	*P*
Strain (*S*)	10	22.08	2.21	0.77	0.71	0.719
Time (*T*)	3	100.31	33.44	3.52	10.70	<0.001
Concentration (*C*)	10	37.97	3.80	1.33	1.22	0.278
*S* × *T*	30	35.33	1.18	1.24	0.38	0.999
*S* × *C*	100	234.8	2.35	8.23	0.76	0.96
*T* × *C*	30	145.96	4.87	5.12	1.56	0.032
*S* × *T* × *C*	300	764.08	2.55	26.78	0.82	0.974

**Table 5:** Multivariate analysis of variance by three-way ANOVA of proliferation kinetics of *S. epidermidis *strains in the presence of EEPP expressed as reduction of AlmarBlue.

Source of variation	df	Sum of squares	Mean squares	% of variance	*F*	*P*
Strain (*S*)	10	24371	2437	3.03	176.4	<0.001
Time (*T*)	4	200813	50203	24.94	3633.5	<0.001
Concentration (*C*)	10	239404	23940	29.73	1732.7	<0.001
*S* × *T*	40	15932	398	1.98	28.8	<0.001
*S* × *C*	100	20566	206	2.55	14.9	<0.001
*T* × *C*	40	233404	5835	28.99	422.3	<0.001
*S* × *T* × *C*	400	70649	177	8.77	12.8	<0.001
